# Globalization and Health: Exploring the opportunities and constraints for health arising from globalization

**DOI:** 10.1186/1744-8603-1-2

**Published:** 2005-04-22

**Authors:** Derek Yach

**Affiliations:** 1 Yale University School of Medicine, Department of Epidemiology and Public Health, 60 College Street, Suite 319, New Haven, CT 06520-8034, USA

## Abstract

The tremendous benefits which have been conferred to almost 5 billion people through improved technologies and knowledge highlights the concomitant challenge of bringing these changes to the 1 billion people living mostly in sub-Saharan Africa and South Asia who are yet to benefit. There is a growing awareness of the need to reduce human suffering and of the necessary participation of governments, non-government organizations and industry within this process. This awareness has recently translated into new funding mechanisms to address HIV/Aids and vaccines, a global push for debt relief and better trade opportunities for the poorest countries, and recognition of how global norms that address food safety, infectious diseases and tobacco benefit all. '*Globalization and Health*' will encourage an exchange of views on how the global architecture for health governance needs to changes in the light of global threats and opportunities.

## Editorial

Over the last 50 years there has been a steady convergence in the probability of survival and the causes of death between the wealthiest countries of the world and most living in low-middle income countries. This represents a triumph for global health and development. It is a demonstration of what should be possible for the 1 billion people living mainly in sub-Saharan Africa and South Asia. For them, convergence seems way off. And survival is seriously compromised by a combination of diseases classically associated with underdevelopment and the more recent emergence of HIV/Aids.

For almost 5 billion people globalization has been associated with increased access to knowledge and technologies that improve life's prospects. The reduction of fatalism has been replaced by a growing global awareness that the "right to health" can become a reality when governments, nongovernmental organizations and industry play their part.

Global awareness among people and governments in wealthiest countries about the need to reduce suffering for all has led to many new initiatives in just the last few years. These include new funding mechanisms to address HIV/Aids and vaccines, a global push for debt relief and better trade opportunities for the poorest countries, and recognition of how global norms that address food safety, infectious diseases and tobacco benefit all.

This journal will encourage debate and dialogue about how progress can be accelerated so as to reduce health differences in survival and quality of life that are amenable to policy and operational interventions. It will also stimulate discussion about how new and continuing threats to all can be prevented through multi-sectoral and international actions.

We will encourage an exchange of views on how the global architecture for health governance needs to changes in the light of global threats and opportunities. Over the relatively short period of the last 5 to 8 years, many new players have emerged on the global scene with additional funding and their own sets of priorities for investing in health and health research. Simultaneously, major United Nations players like the WHO, World Bank and UNICEF have changed their focus. We will stimulate discussion about how best to enhance the prospects of improving health at the local and national level through better global governance, and hope for an exchange of ideas that will be innovative and helpful to the process of improving global health.

## Competing interests

There are no competing interests related to this work. But in the interests of full disclosure, Derek Yach is funded at Yale to develop community based chronic disease prevention research models internationally by Novo Nordisk.

